# Soil Carbon and Microbial Functioning Reveal Multidimensional Patterns Across a *Cedrus libani* Afforestation Chronosequence in Mediterranean Karst

**DOI:** 10.3390/biology15181594

**Published:** 2026-09-10

**Authors:** Emre Babur, Burak Yalçıntaş, Yasin Taha Ünsal, Uğur Kezik, Bülent Akgün, Emre Yazar

**Affiliations:** 1 Department of Forest Engineering, Kahramanmaraş Sütçü İmam University, 46050 Kahramanmaraş, Türkiye; burak3379@gmail.com (B.Y.); tahayasin4664@gmail.com (Y.T.Ü.); ugurkezik@ksu.edu.tr (U.K.); bakgun@ksu.edu.tr (B.A.); 2 Department of Bioengineering and Sciences, Kahramanmaraş Sütçü İmam University, 46050 Kahramanmaraş, Türkiye; emre.yazar6@gmail.com

**Keywords:** ecological restoration, soil organic carbon, microbial biomass carbon, metabolic quotient, soil health, restoration monitoring

## Abstract

Afforestation assessments in Mediterranean karst regions often focus on vegetation and soil carbon, paying less attention to microbial activity. We examined the surface soil properties of a four-site *Cedrus libani* chronosequence in southern Turkey. In October 2024, topsoil (0–10 cm deep) was sampled at one degraded reference site and three plantation sites aged approximately 9, 14 and 24 years (15 samples were taken from each site). The afforested sites generally exhibited higher levels of soil organic carbon, total nitrogen, aggregate stability and microbial biomass carbon (MBC). Mean MBC ranged from 288 to 536 mg C kg^−1^ at afforested sites versus 180 mg C kg^−1^ at the reference site. Basal respiration was lower (0.30–0.36 versus 0.96 µg CO_2_-C g^−1^ h^−1^), as was biomass-specific respiration (*q*CO_2_: 0.54–1.39 versus 5.41 mg CO_2_-C g^−1^ MBC h^−1^). The microbial quotient did not differ significantly. The pooled MBC–respiration association was weak, whereas the site-adjusted association was positive. Multivariate patterns and the non-monotonic ordering of sites highlighted edaphic heterogeneity. As plantation age was completely confounded with site identity, these single-campaign observations cannot establish causal temporal effects. Nevertheless, integrating structural, chemical and microbial indicators provides complementary information for characterizing soil conditions across these karst sites.

## Simple Summary

Planting trees is widely used to restore degraded Mediterranean landscapes, but tree establishment alone does not show whether soil conditions have improved. We compared surface soils from a degraded reference site and three cedar plantations of different ages in a limestone landscape in southern Turkey. The plantation sites generally contained higher levels of organic carbon, nitrogen and microbial biomass, and their soil aggregates were more stable. However, laboratory respiration was lower per unit of soil and per unit of microbial biomass. These differences were not simply related to plantation age, and the sites also differed in their underlying soil properties. As only one site was sampled for each age class and sampling occurred once, we cannot determine whether planting trees caused the observed differences. Our findings emphasize the importance of examining soil structure, chemistry and microbial measurements together, and highlight the need for repeated measurements and independently replicated sites to assess restoration over time.

## 1. Introduction

Afforestation is increasingly used as a nature-based intervention to rehabilitate degraded landscapes, enhance soil carbon sequestration, reduce erosion, and improve ecosystem resilience. However, successful restoration cannot be inferred from tree establishment alone because recovery of belowground functions may lag behind vegetation development. Soil structure, nutrient cycling, carbon stabilization, and microbial functioning collectively determine whether restored vegetation translates into persistent improvements in ecosystem condition [1,2,3]. Therefore, assessing restoration requires an integrated evaluation of the physical, biogeochemical and microbial processes that recover together, rather than relying on changes in individual soil properties.

Mediterranean karst landscapes represent an exceptionally complex setting for functional recovery. Karst terrain covers approximately 12% of the global land surface and is defined by thin, spatially discontinuous soils overlaying carbonate bedrock [4,5]. Soil primarily accumulates within dissolution pockets, bedrock fissures, and residual mantles; however, shallow profiles, high skeleton content, low organic matter, and limited water-holding capacity severely restrict soil development and vegetation establishment [6,7,8]. Given that carbonate weathering produces minimal insoluble residue, mineral soil formation progresses extremely slowly, thereby amplifying the critical role of organic matter in maintaining soil structure, nutrient retention, and water storage [9,10]. Consequently, vegetation removal and the subsequent loss of organic matter and aggregate stability can drive these fragile ecosystems into severe, irreversible degradation. These severe edaphic constraints underscore the imperative for site-specific indicators capable of decoupling true functional recovery from ambient soil variability during ecological restoration.

Developing tree canopies modify soil temperature and moisture regimes, while litter inputs, root turnover, and rhizosphere activity increase organic matter inputs and nutrient cycling [11,12,13,14]. Increasing organic inputs can promote aggregate formation and stabilization, which in turn physically protect newly incorporated organic matter from rapid mineralization [15,16,17]. Improved soil structure may further enhance water retention and nutrient conservation, creating positive feedbacks between vegetation development, carbon accumulation, and soil biological activity. Consequently, belowground differences associated with afforestation should be evaluated as coordinated changes in soil structural, biogeochemical, and biological properties rather than solely as changes in SOC. The microbial compartment provides a particularly sensitive link between organic matter inputs and their stabilization. Soil microorganisms simultaneously decompose plant-derived substrates, immobilize nutrients, and contribute microbial residues to persistent SOC [18,19,20]. Among potential monitoring indicators, MBC and basal respiration provide complementary measures of microbial pool size and carbon mineralization, whereas *q*CO_2_ expresses respiration relative to microbial biomass and can indicate changes in microbial maintenance demand [21,22,23,24]. Together with aggregate stability and SOC, these variables can therefore distinguish increases in carbon pools from changes in the functioning of the soil system [12,25].

This distinction is particularly relevant in degraded karst soils, where high surface temperatures, episodic moisture availability, and limited substrate supply may impose substantial metabolic costs on microbial communities. Following tree establishment, increased organic inputs and improved microclimatic conditions could increase microbial biomass while simultaneously reducing respiration per unit biomass. Under such a trajectory, restoration would not necessarily be reflected by a greater proportion of SOC held in microbial biomass, but rather by a decline in microbial respiratory carbon loss relative to the size of the microbial pool. Integrating SOC, nutrient status, soil structure, microbial biomass, basal respiration, *q*Mic, and *q*CO_2_ can therefore provide a more process-oriented assessment of soil recovery than any of these indicators considered independently.

Chronosequence approaches offer a practical framework for examining such long-term trajectories where continuous monitoring is unavailable. By substituting space for time, stands of different ages can reveal decadal changes in soil carbon, nutrient cycling, structure, and microbial functioning following afforestation [14,26,27]. However, this approach is particularly challenging in karst landscapes because pronounced pedodiversity can occur over short distances. Soil pockets may differ in depth, residual mineral material, carbonate content, and pH even under similar climate and topography [9]. Consequently, apparent stand-age effects may partly reflect pre-existing edaphic differences rather than ecosystem development alone. Explicitly accounting for this heterogeneity is therefore necessary before temporal changes observed along a karst chronosequence can be attributed to afforestation.

*Cedrus libani* A. Rich. (Taurus cedar) is a native conifer of the eastern Mediterranean and one of the principal species used for afforestation of degraded high-elevation karst landscapes in southern Türkiye because of its drought tolerance, deep rooting system, and ability to establish on shallow, rocky, calcareous substrates [28,29]. A previous study by Babur et al. [30] examined soil organic carbon, total nitrogen, bulk density, and derived C and N stocks at the degraded reference site and the 9- and 24-year-old *C. libani* plantations included in the present chronosequence. Those data were obtained from the same October 2024 sampling campaign used here. The present study extends that earlier work by including an additional 14-year-old plantation and, more importantly, by addressing a different research question: whether biogeochemical differences among chronosequence sites are accompanied by coordinated changes in soil structure and microbial functioning under pronounced edaphic heterogeneity. Accordingly, the principal new contribution of the present study is the integration of aggregate stability, microbial biomass carbon, basal respiration, *q*Mic, and *q*CO_2_ with edaphic and multivariate analyses, rather than a re-analysis of soil C and N stocks. However, carbon accumulation alone does not establish whether belowground ecosystem functioning is recovering. It remains unclear whether changes in soil carbon are accompanied by coordinated improvements in soil structure and microbial metabolism, whether microbial ecophysiological indicators respond differently from bulk soil properties, and to what extent these patterns can be distinguished from underlying variation in carbonate-rich parent material.

Despite widespread use of afforestation in degraded Mediterranean landscapes, restoration monitoring still relies heavily on vegetation establishment and changes in individual soil properties. Less is known about whether structural, biogeochemical, and microbial indicators provide consistent evidence of functional recovery when restoration sites differ in underlying edaphic conditions. This distinction is particularly important in karst terrain, where strong small-scale variation in carbonate content, soil depth, and parent material can obscure or amplify apparent restoration responses.

Accordingly, this study evaluated patterns in surface-soil functioning across a four-site *C. libani* afforestation chronosequence in degraded Mediterranean karst of southern Türkiye. We integrated structural, biogeochemical, and microbial indicators and explicitly evaluated whether observed responses remained robust to variation in carbonate content and soil pH. We hypothesized that (i) afforested sites would exhibit greater aggregate stability and higher SOC, TN, and MBC than the degraded reference site; (ii) microbial metabolic indices would differ between the degraded reference and afforested sites, particularly through lower biomass-specific respiration; and (iii) structural and microbial properties would be less sensitive to edaphic heterogeneity than SOC and TN. By testing these hypotheses, we aimed to identify soil indicators that can improve monitoring of afforestation success and inform site-specific restoration management in environmentally heterogeneous karst landscapes. Thus, whereas the previous study [30] focused on the magnitude of soil C and N pools, the present study tests whether those biogeochemical patterns are coupled to soil structural condition and microbial metabolic functioning and evaluates the extent to which these integrated relationships are sensitive to the pronounced edaphic heterogeneity of Mediterranean karst.

## 2. Materials and Methods

### 2.1. Study Area

**Figure 1:**
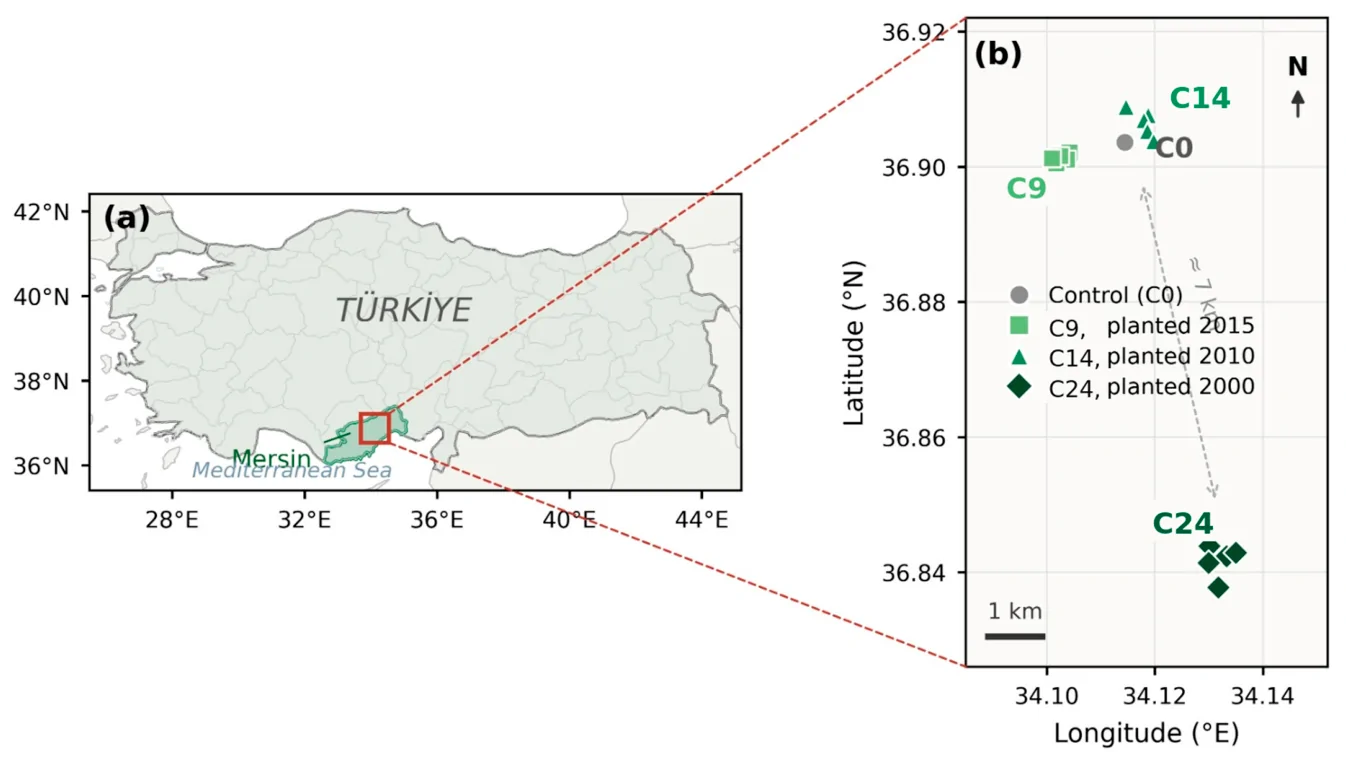
Location of the study sites. (**a**) Position of Mersin Province within Türkiye, with the study area boxed. (**b**) The four sampling areas in the upland part of Erdemli District: unafforested reference site (C0) and *Cedrus libani* plantations established in 2015 (C9), 2010 (C14) and 2000 (C24). Symbols mark the recorded cylinder sampling points; the C24 site lies approximately 7 km south of the other three. Coordinates were recorded in UTM zone 36 N and converted to geographic coordinates.

The study was conducted in *C. libani* A. Rich. afforestation sites in the Sorgun–Toros highlands of Erdemli District, Mersin Province, southern Türkiye (36°50′–36°54′ N, 34°06′–34°08′ E; Figure 1). The sites are located on the southern flank of the Central Taurus Mountains. The four study sites occurred within an elevation range of 1655–1690 m a.s.l.; however, they also differed in slope and several edaphic properties, as described below. The three plantations were established in 2015, 2010, and 2000, respectively. Because soil sampling was conducted in October 2024, these stands were approximately 9, 14, and 24 years old at the time of sampling and are hereafter designated C9, C14, and C24. The non-afforested degraded reference site is designated C0. Stand age is used as a nominal chronosequence descriptor rather than as an independently replicated temporal treatment. The 9-year, 14-year, and reference sites occur within approximately 1.5 km of one another, whereas the 24-year plantation is located approximately 7 km farther south. Geographic coordinates and stand characteristics are presented in Table 1.

Because the nearest long-term meteorological station at Erdemli is located approximately 35 km south-southeast of the study area and about 1660 m lower in elevation, its records were considered unrepresentative of the high-elevation study sites. Climatic conditions were therefore characterized using WorldClim 2.1 climate surfaces at 30-arc-second spatial resolution, extracted for each site. Mean annual temperature ranges from 8.5 to 9.0 °C and annual precipitation from 556 to 574 mm across the study area. The sites experience pronounced Mediterranean seasonality, with cold winters and a marked summer drought from June to September; June–August precipitation totals only approximately 39–41 mm. Climatic variation among the four sites is minor relative to the strong seasonal water limitation characteristic of the upper Mediterranean karst landscape.

The soils are predominantly terra rossa developed on limestone-derived karstic parent materials [31]. Geologically, the area lies within the Taurus orogenic belt, where sedimentary formations from the Oligocene and Pliocene periods lie on top of Palaeozoic and Mesozoic basement rocks in an unconformable manner [32]. Limestone outcrops, dissolution cavities, and shallow discontinuous soil pockets are widespread, producing pronounced small-scale edaphic heterogeneity typical of Mediterranean karst terrain [33]. These soils generally have limited effective depth and water-holding capacity, conditions that strongly constrain vegetation establishment and soil development.

*C. libani* is the dominant native conifer at these elevations and has been extensively used for restoration of degraded karst lands in the region. All plantations were established by the General Directorate of Forestry using comparable operational afforestation practices, including similar site preparation and planting procedures. No fertilization, grazing, or soil amendments were applied after plantation establishment. Although the oldest plantation is spatially separated from the younger stands, all sites occur within the same high-elevation Mediterranean karst landscape and experience closely comparable climatic conditions and broadly similar soil-forming environments. Residual differences in soil carbonate content and pH among sites were therefore treated explicitly in the statistical analyses rather than assuming complete edaphic equivalence across the chronosequence. Mean slope also differed among sites (10–40%), and this topographic variation may influence runoff, erosion, soil-water redistribution, and organic-matter accumulation. Because effective soil depth, coarse-fragment abundance, and microsite water availability were not quantified at each sampling location, these factors could not be included directly in the statistical adjustment and are treated as potential sources of residual site-level heterogeneity.

### 2.2. Experimental Design

An observational space-for-time chronosequence was used to characterize differences in soil properties and belowground functioning among a degraded reference site and three *C. libani* plantation sites of different nominal ages. Three plantations established in 2015, 2010, and 2000 were selected to represent successive stages of stand development and are hereafter referred to as C9, C14, and C24, respectively. Adjacent non-afforested degraded land (C0) was used as a reference site for comparison; in the absence of historical baseline measurements, it was not assumed to represent the actual pre-afforestation condition of all plantations. The 15 sampling locations within each class characterize within-site variability and were not treated as independent stand-level replicates. Plantation age was therefore interpreted only as a nominal chronosequence descriptor, and all observed differences were evaluated as site- or chronosequence-associated patterns together with potential edaphic variation.

The sites were selected to be comparable in elevation, climatic setting, topography, broad soil-forming environment, and previous land-use history. All sites occur within the same high-elevation Mediterranean karst landscape and are associated with limestone-derived terra rossa soils. Although the oldest plantation was located in a different forest enterprise, the sites were selected to minimize environmental variation unrelated to stand development. Nevertheless, complete site equivalence cannot be assumed in a space-for-time design, particularly in heterogeneous karst terrain. Residual edaphic variation was explored statistically using soil CaCO_3_ content and pH as covariates in sensitivity analyses. These analyses were not intended to isolate a causal stand-age effect, because other potentially important site characteristics, including slope, effective soil depth, coarse-fragment abundance, and microsite water availability, were not quantified comprehensively and could not be statistically controlled.

The experimental design comprised four chronosequence classes (C0, C9, C14, and C24). Fifteen spatially distributed sampling locations were established within each class, yielding 60 sampling locations in total. Sampling locations were distributed to maximize spatial coverage within the available plantation and reference area and to reduce local spatial dependence. Plantation age was used only as a nominal chronosequence descriptor; it was not treated as an independently replicated temporal factor.

Forest structural characteristics were determined in three 10 m × 10 m plots within each plantation. Diameter at breast height (DBH) and total tree height were measured for all stems within each plot, resulting in inventories of 60, 61, and 48 trees in C9, C14, and C24, respectively. Stand basal area was calculated from individual stem diameters, and stem density was estimated from tree counts within the measured plots.

### 2.3. Soil Sampling

Soil sampling was conducted in October 2024 at the 60 sampling locations distributed across the four chronosequence sites/classes. Before sampling, the surface litter layer was carefully removed to expose the mineral soil. At each location, five soil cores were collected from the 0–10 cm mineral layer, one from the center and four from the cardinal directions, and combined to form a single composite sample. The 0–10 cm layer was selected because it represents the biologically active surface soil most responsive to changes in organic matter inputs, root activity, and microbial processes following afforestation.

Separate samples were collected for physicochemical and microbial analyses. In addition, an undisturbed soil core was obtained at each sampling location using a 100 cm^3^ steel cylinder for determination of bulk density. The geographic coordinates of all sampling locations were recorded. Soil sampling followed the International Co-operative Programme on Assessment and Monitoring of Air Pollution Effects on Forests (ICP Forests) guidelines for soil horizon and sampling depth, while the Area-Frame Randomised Soil Sampling approach was used to guide the spatial distribution of sampling locations [34,35,36].

Samples intended for physicochemical analyses were air-dried, visible roots and plant residues were removed, and the soil was passed through a 2-mm sieve before analysis. Results were expressed on an oven-dry, <2-mm fine-earth basis unless otherwise stated. Samples intended for microbial analyses were maintained at field moisture, placed in sealed polyethylene bags, transported to the laboratory under cooled conditions, stored at 4 °C immediately after collection, and analyzed within 48 h to minimize changes in microbial activity during storage.

The C0, C9, and C24 samples were collected during the same October 2024 field campaign used in our previous study [30]. For these three sites, SOC, TN, and bulk-density measurements therefore overlap with the previously published dataset. The present study additionally includes the C14 site and new measurements of aggregate stability and microbial attributes, which were integrated to address soil functional patterns rather than C and N stock quantification alone.

### 2.4. Laboratory Analyses

All laboratory analyses were conducted at the Soil Science Laboratory of Kahramanmaraş Sütçü İmam University using internationally accepted standard methods. Soil physicochemical and microbial properties were determined according to the analytical procedures described below.

#### 2.4.1. Physicochemical Analyses

Gravimetric soil water content was determined by drying field-moist soil samples at 105 °C to constant mass. Soil particle-size distribution (sand, silt, and clay) was determined using the hydrometer method [37]. Soil texture classes were assigned according to the USDA Soil Texture Classification System. Soil pH and electrical conductivity (EC) were measured in a 1:2.5 (*w*/*v*) soil-to-distilled water suspension. The pH meter was calibrated before each analytical batch using standard buffer solutions bracketing the sample pH range, and the conductivity meter was checked using a certified conductivity standard. Electrodes were rinsed with distilled water between samples. Measurements were conducted in duplicate, and the mean value was used for analysis. EC was expressed as dS m^−1^ at 25 °C.

SOC was determined using the Walkley–Black wet oxidation method [38], whereas total nitrogen (TN) was measured using Kjeldahl digestion [39]. Total calcium carbonate (CaCO_3_) was determined volumetrically using a Scheibler calcimeter [39].

Bulk density (BD) was determined following Blake and Hartge [40]: undisturbed cores taken with 100 cm^3^ steel cylinders were oven-dried at 105 °C for 24 h, weighed, and bulk density calculated as the oven-dry mass divided by the cylinder volume. Coarse-fragment (>2 mm) volume and the corresponding fine-earth fraction were not determined, and effective soil depth was not recorded systematically at each sampling location. Area-based soil C and N stocks are therefore not reported in this study because uncorrected stock estimates are prone to overestimation in stony karst soils; all carbon and nitrogen results are expressed as fine-earth concentrations.

For C0, C9, and C24, SOC, TN, and bulk-density values derive from the same sample set previously reported in Babur et al. [30]. These variables are retained here only as biogeochemical and physical context for integration with the newly evaluated structural and microbial indicators. Measurements from C14 were generated as part of the expanded four-site chronosequence used in the present study.

Aggregate stability (AS) was determined using the wet-sieving method and expressed as the percentage of water-stable aggregates described by Kemper and Rosenau [41], with minor modifications as applied to eroded and restored soils of the region by Babur et al. [42]. For this, air-dried soil samples (1.0–2.0 mm size fraction, m soil = 4.0 g) were placed on a 0.25 mm sieve. Samples were pre-wetted by capillary action by placing the sieves on moist filter paper for 10–15 min to minimize slaking from sudden air entrapment. The wetted samples were then submerged in deionized water and stroke-oscillated (vertical stroke length of 1.3 cm at 30 cycles min^−1^) using a wet-sieving apparatus for 5 min. After oscillation, the soil remaining on the sieve was rinsed into an evaporating dish and dried at 105 °C for 24 h to determine the weight of water-stable aggregates (Mwsa). To correct for coarse sand grains (>0.25 mm), the retained aggregates were dispersed in a 0.5% sodium hexametaphosphate (NaPO_3_)_6_ solution, and washed through the same 0.25-mm sieve, and the weight of the remaining sand fraction (Msand) was subtracted. Aggregate stability (AS, expressed as %) was calculated using the following Formula (1):

(1) $$AS\left(\%\right)=\left(\frac{Mwsa-Msand}{Msoil-Msand}\right)\times \;100$$

where: *Mwsa* = dry mass of soil retained on the 0.25-mm sieve after wet sieving (g), *Msand* = dry mass of sand grains retained on the 0.25-mm sieve after dispersion (g), *Msoil* = initial dry mass of the soil sample (g).

#### 2.4.2. Microbial Biomass Carbon, Basal Respiration, and Microbial Quotients

Microbial biomass carbon (MBC) was determined using the chloroform fumigation–extraction (CFE) method following the standardized protocol of Horwath and Paul [43], based on the original procedures of Brookes et al. [44] and Vance et al. [45]. For each sample, two fresh subsamples (30 g) were prepared. One subsample was fumigated with ethanol-free chloroform in a vacuum desiccator at 25 °C for 24 h, whereas the second subsample remained unfumigated as the control. Both fumigated and unfumigated soils were extracted with 0.5 M K_2_SO_4_ at a 1:4 (*w*/*v*) soil-to-extractant ratio by shaking at 200 rpm for 30 min. After filtration through Whatman No. 42 filter paper, extractable organic carbon was determined using the Walkley–Black procedure described above. MBC was expressed as mg C kg^−1^ oven-dry soil. MBC was calculated as MBC = 2.64 × *EC*, where *EC* is the difference in extractable organic C between fumigated and unfumigated soil samples. An extraction-efficiency coefficient (k*_EC_*) of 0.38 was used, equivalent to MBC = 2.64 × *EC*, following the chloroform fumigation–extraction approach of Vance et al. [45]. This coefficient accounts for the fraction of microbial biomass C released and recovered during fumigation and K_2_SO_4_ extraction.

Basal respiration (BR) was determined by alkali trapping during laboratory incubation [46]. Fresh soil equivalent to 30 g oven-dry mass was placed in airtight 500-mL glass incubation jars, and soil moisture was standardized to 50–60% of water-holding capacity before incubation. Consequently, samples were not incubated at their original field moisture. No separate pre-incubation period was applied after moisture adjustment. Each jar contained a small open beaker with 10 mL of 0.1 M NaOH to trap the CO_2_ evolved during incubation. The jars were sealed and incubated in the dark at 25 °C for 7 days (168 h). Identical jars containing NaOH but no soil were incubated simultaneously and used as blanks. At the end of the incubation period, the NaOH solution was recovered, BaCl_2_ was added to precipitate carbonate, and the remaining alkali was back-titrated with 0.05 M HCl. The amount of CO_2_ evolved was calculated from the difference in acid consumption between blank and soil-containing jars, converted to CO_2_–C, corrected for oven-dry soil mass and incubation time, and expressed as µg CO_2_–C g^−1^ dry soil h^−1^. All measurements were performed in duplicate for each soil sample, and the mean of the two analytical determinations was used in subsequent analyses. Soil-free jars served as procedural blanks, and all respiration values were blank-corrected.

The microbial quotient (*q*Mic) was calculated as the proportion of microbial biomass carbon relative to soil organic carbon:

*q*Mic (%) = (MBC/SOC) × 100 (2)

where MBC is microbial biomass carbon and SOC is soil organic carbon, both expressed on the same mass basis. *q*Mic was expressed as a percentage and represents the fraction of SOC present as microbial biomass carbon.

The metabolic quotient (*q*CO_2_) was calculated as the ratio of basal respiration to microbial biomass carbon:

*q*CO_2_ = (BR/MBC) × 1000 (3)

where BR is expressed as µg CO_2_–C g^−1^ dry soil h^−1^ and MBC as µg C g^−1^ dry soil (numerically equivalent to mg C kg^−1^ dry soil). *q*CO_2_ was therefore expressed as mg CO_2_–C g^−1^ MBC h^−1^ and was interpreted as an operational index of biomass-specific respiration under the standardized incubation conditions [47].

### 2.5. Statistical Analysis

The chronosequence comprised four sites/classes (C0, C9, C14, and C24), with 15 spatially distributed soil samples per site/class (*n* = 60). Each sampling location was the observational unit for within-site characterization; the four sites, not the 15 samples within each site, define the scope of chronosequence inference. The analytical dataset used sample-level 0–10 cm values.

Positively skewed concentration and ratio variables were log_10_-transformed as specified before group testing; pH, particle-size fractions, aggregate stability, and bulk density were analyzed on their original scales. Residual normality was assessed with Shapiro–Wilk and variance homogeneity with Brown–Forsythe tests. Variables meeting parametric assumptions were analyzed by one-way ANOVA followed by Tukey’s HSD; variables failing residual normality were analyzed by Kruskal–Wallis followed by Dunn tests with Holm adjustment. Effect sizes were omega squared (ω^2^) for ANOVA and epsilon squared (ε^2^) for Kruskal–Wallis. The 15 omnibus *p* values were adjusted as one Benjamini–Hochberg FDR family; both raw and FDR-adjusted values were cross-checked against the same final model for each variable.

Multivariate differences among sites/classes were evaluated by PERMANOVA using 9999 permutations and Euclidean distances. The matrix comprised two texture ilr coordinates plus bulk density, aggregate stability, pH, EC, CaCO_3_, SOC, TN, MBC, and BR. EC, CaCO_3_, SOC, TN, MBC, and BR were log_10_-transformed, after which all columns were z-standardized. C/N, *q*Mic, and *q*CO_2_ were excluded from the multivariate matrix to avoid duplicate weighting of their components. PERMDISP used 4999 permutations. PCA was fitted to the same corrected 11-variable matrix.

Pairwise associations among 15 variables were evaluated by Spearman rank correlation. For site/class-adjusted partial Spearman analysis, each variable was converted to midranks and separately regressed on an intercept plus three indicator covariates (C9, C14, and C24; C0 reference); Pearson correlation of the two residual vectors yielded the partial coefficient. Significance used t = r√[(n − k − 2)/(1 − r^2^)] with *n* = 60, k = 3, and df = 55. BH–FDR correction was applied across 105 pairs. Correlations involving C/N, *q*Mic, *q*CO_2_, or their components were treated cautiously because mathematical coupling can create or strengthen associations; sand–silt–clay correlations were considered descriptive because of compositional closure. Exploratory ANCOVA sensitivity models used site/class, pH, and measured CaCO_3_, followed by joint tests of all six site/class × covariate interactions. Common-slope outputs were not interpreted when this interaction test was significant. Collinearity was assessed using Pearson and Spearman correlations, variance-inflation factors (VIFs), and the standardized-design condition number. All statistical analyses were performed in Python 3.12.4 (Python Software Foundation, Wilmington, DE, USA) using NumPy 2.1.1, pandas 2.2.2, SciPy 1.14.1, statsmodels 0.14.2, scikit-posthocs 0.9.0 and scikit-bio 0.6.2; figures were produced with Matplotlib 3.9.2.

## 3. Results

Soil physicochemical and microbial properties differed markedly among the four chronosequence sites/classes. Fourteen of the 15 omnibus tests remained significant after BH–FDR correction. *q*Mic was the only non-significant outcome; for the prespecified log_10_-scale ANOVA, raw *p* = 0.0801 and BH–FDR *p* = 0.0801.

The largest standardized site/class differences were observed for *q*CO_2_ (ω^2^ = 0.865), CaCO_3_ (ω^2^ = 0.640), pH (ε^2^ = 0.620), BR (ω^2^ = 0.566), TN (ε^2^ = 0.544), EC (ε^2^ = 0.532), and aggregate stability (ε^2^ = 0.510). MBC showed ω^2^ = 0.337, whereas *q*Mic showed a small and non-significant effect (ω^2^ = 0.064).

### 3.1. Site-Associated Variation in Soil Physicochemical Properties

**Figure 2:**
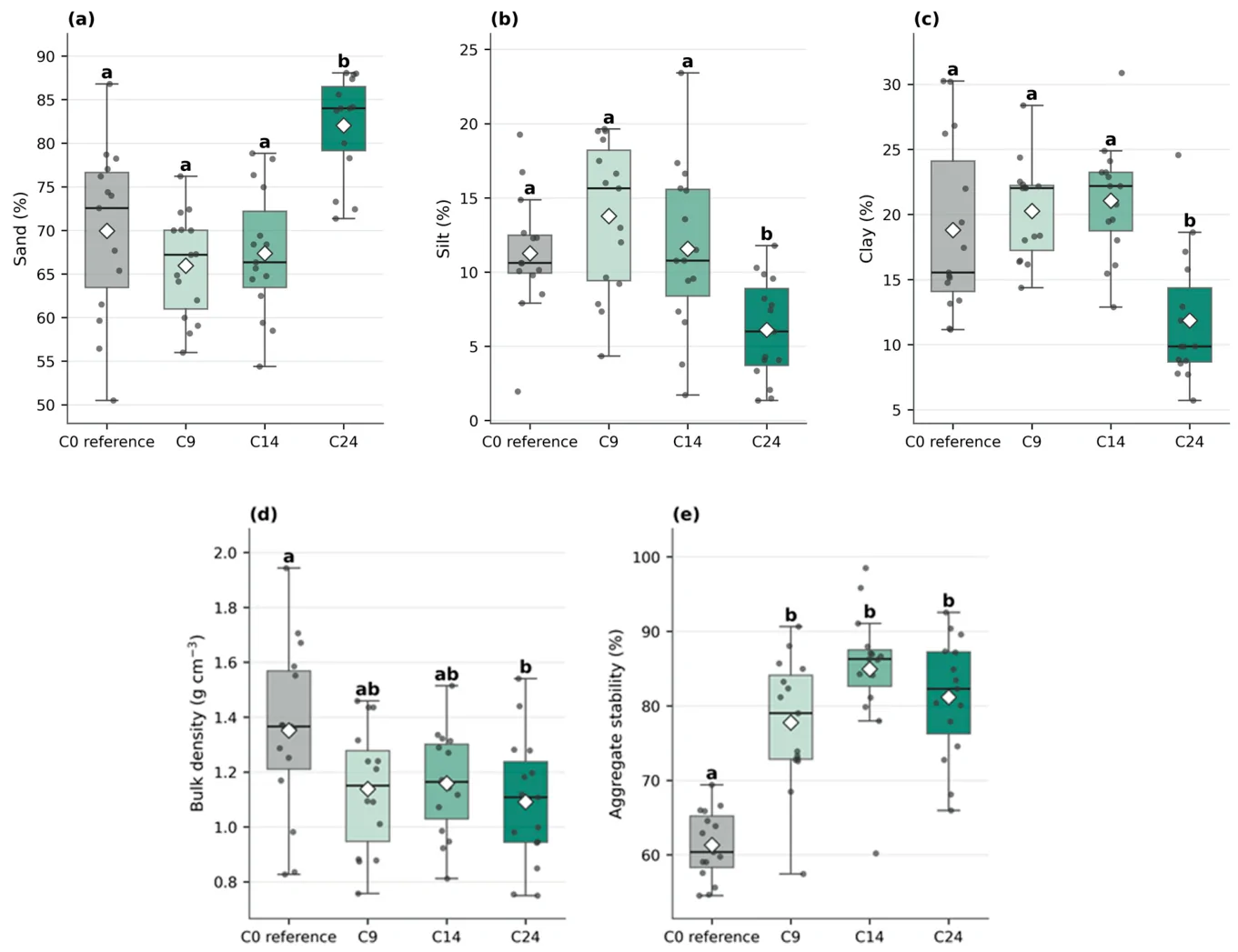
Soil particle-size distribution and physical properties across the four chronosequence classes: (**a**) sand, (**b**) silt, (**c**) clay, (**d**) bulk density, and (**e**) aggregate stability. Boxes indicate the interquartile range, horizontal lines within the boxes represent medians, white diamonds show arithmetic means, and jittered points represent individual samples (*n* = 15 per class). Whiskers extend to the most extreme observations within 1.5 times the interquartile range. Compact-letter displays are centered immediately above the upper whisker caps. Classes sharing at least one lowercase letter do not differ significantly, whereas classes with no letter in common differ at *p* < 0.05. Pairwise comparisons were performed using Tukey’s HSD following one-way ANOVA for sand, silt, and bulk density, and Dunn’s test with Holm adjustment following the Kruskal–Wallis test for clay and aggregate stability.

**Figure 3:**
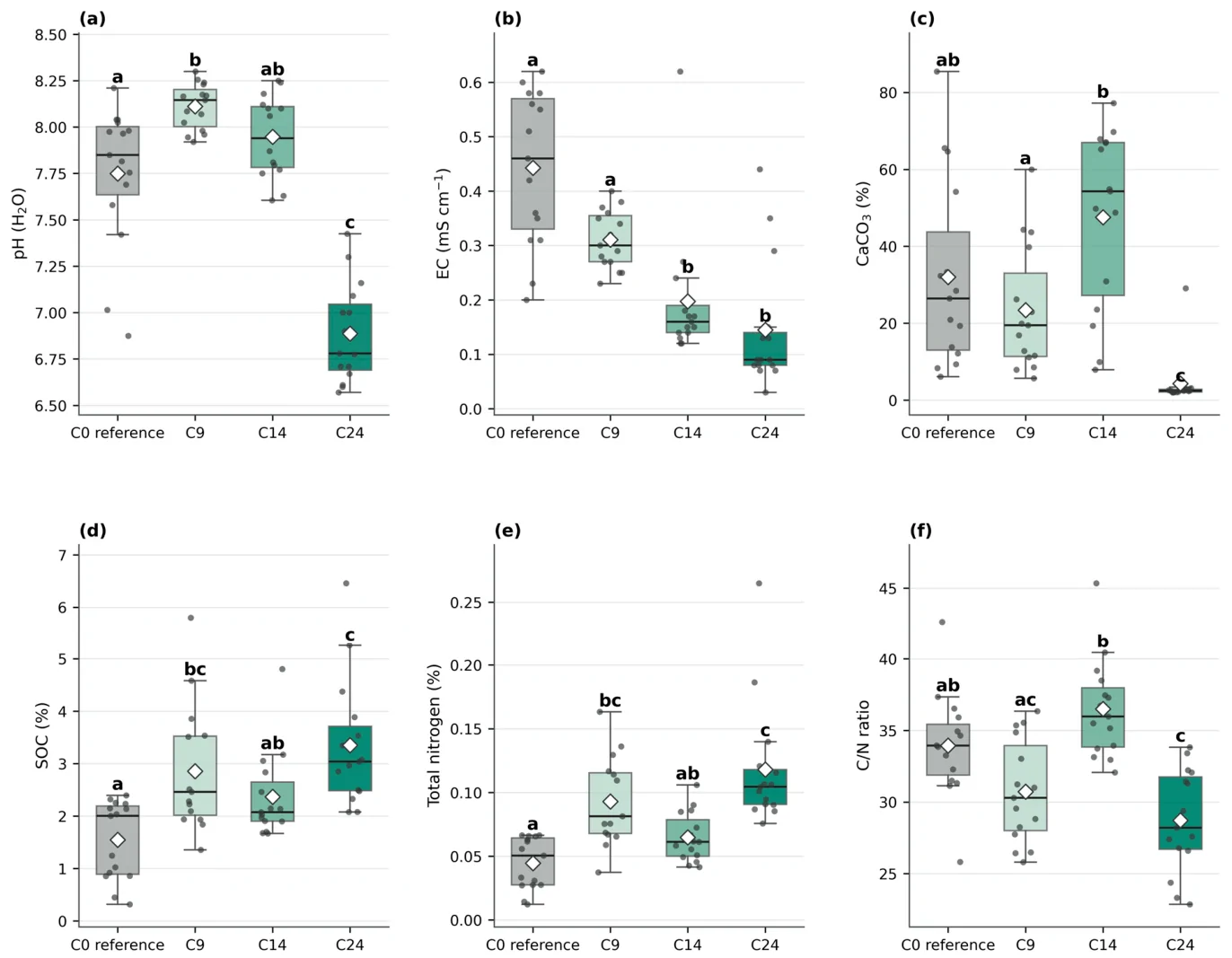
Soil chemical properties across the four chronosequence classes: (**a**) pH, (**b**) electrical conductivity (EC), (**c**) calcium carbonate (CaCO_3_), (**d**) soil organic carbon (SOC), (**e**) total nitrogen (TN) for the 0–10 cm soil layer, and (**f**) the recalculated C/N ratio. Boxes represent the interquartile range, horizontal lines indicate medians, white diamonds show arithmetic means, and jittered points represent individual samples (*n* = 15 per class). Whiskers extend to the most extreme observations within 1.5 times the interquartile range. Compact-letter displays are centered immediately above the upper whisker caps. Classes sharing at least one lowercase letter do not differ significantly, whereas classes with no letter in common differ at *p* < 0.05. Tukey’s HSD following one-way ANOVA was used for CaCO_3_ and C/N, whereas Dunn’s test with Holm adjustment following the Kruskal–Wallis test was used for pH, EC, SOC, and TN. Statistical analyses of EC, CaCO_3_, SOC, TN, and C/N were performed using log_10_-transformed values, while the figure presents the original measurement scales.

Surface-soil physicochemical properties differed substantially among the *C. libani* chronosequence sites (Table 2; Figure 2 and Figure 3). Following Benjamini–Hochberg false discovery rate (FDR) correction, all physicochemical variables remained significantly different among sites/classes, indicating consistent differences among chronosequence sites. Effect sizes ranged from moderate to very large, with the strongest responses observed for calcium carbonate (ω^2^ = 0.640), soil pH (ε^2^ = 0.620), TN (ε^2^ = 0.544), EC (ε^2^ = 0.532), and AS (ε^2^ = 0.510).

#### 3.1.1. Soil Physical Properties

Particle-size distribution differed among sites/classes, principally because C24 had a distinct texture (Figure 2). Sand (ANOVA, *p* < 0.001, ω^2^ = 0.409) and silt (ANOVA, *p* < 0.001, ω^2^ = 0.243) met parametric assumptions, whereas clay was analyzed by Kruskal–Wallis (*p* < 0.001, ε^2^ = 0.307). C0, C9, and C14 did not differ significantly in any fraction; C24 had higher sand and lower silt and clay.

Bulk density differed among sites/classes (ANOVA, raw *p* = 0.0275, BH–FDR *p* = 0.0295, ω^2^ = 0.102). Tukey’s HSD detected a difference between C0 and C24 (*p* = 0.0260), while C9 and C14 were intermediate and did not differ from either endpoint. Aggregate stability showed a much larger difference (Kruskal–Wallis, *p* < 0.001, ε^2^ = 0.510): C0 was lower than each afforested site, whereas C9, C14, and C24 did not differ significantly from one another.

#### 3.1.2. Soil Chemical Properties

Soil pH differed among sites/classes (Kruskal–Wallis, *p* < 0.001, ε^2^ = 0.620). C9 had the highest values; C0 and C14 shared a compact-letter group; and C24 was lower than each of the other three sites/classes.

EC differed among sites/classes (Kruskal–Wallis, *p* < 0.001, ε^2^ = 0.532). C0 and C9 did not differ significantly, C14 and C24 did not differ significantly, and the latter two sites had lower EC than C0; C9 also differed from C14 and C24.

SOC differed among sites/classes (Kruskal–Wallis, *p* < 0.001, ε^2^ = 0.313), with C24 exceeding C0 and C14 but not C9. TN also differed (Kruskal–Wallis, *p* < 0.001, ε^2^ = 0.544): C24 exceeded C0 and C14, C9 exceeded C0, and C9 did not differ from C14 or C24 after Dunn–Holm adjustment. C/N differed by log_10_-scale ANOVA (*p* < 0.001, ω^2^ = 0.391), with C14 higher than C9 and C24. CaCO_3_ differed by log_10_-scale ANOVA (*p* < 0.001, ω^2^ = 0.640); C24 was lower than all other sites/classes; while C9 was lower than C14.

Overall, chemical properties differed markedly among the chronosequence sites/classes, with the largest differences observed for soil pH, CaCO_3_, TN, and EC.

### 3.2. Site-Associated Variation in Soil Microbial Functioning

**Figure 4:**
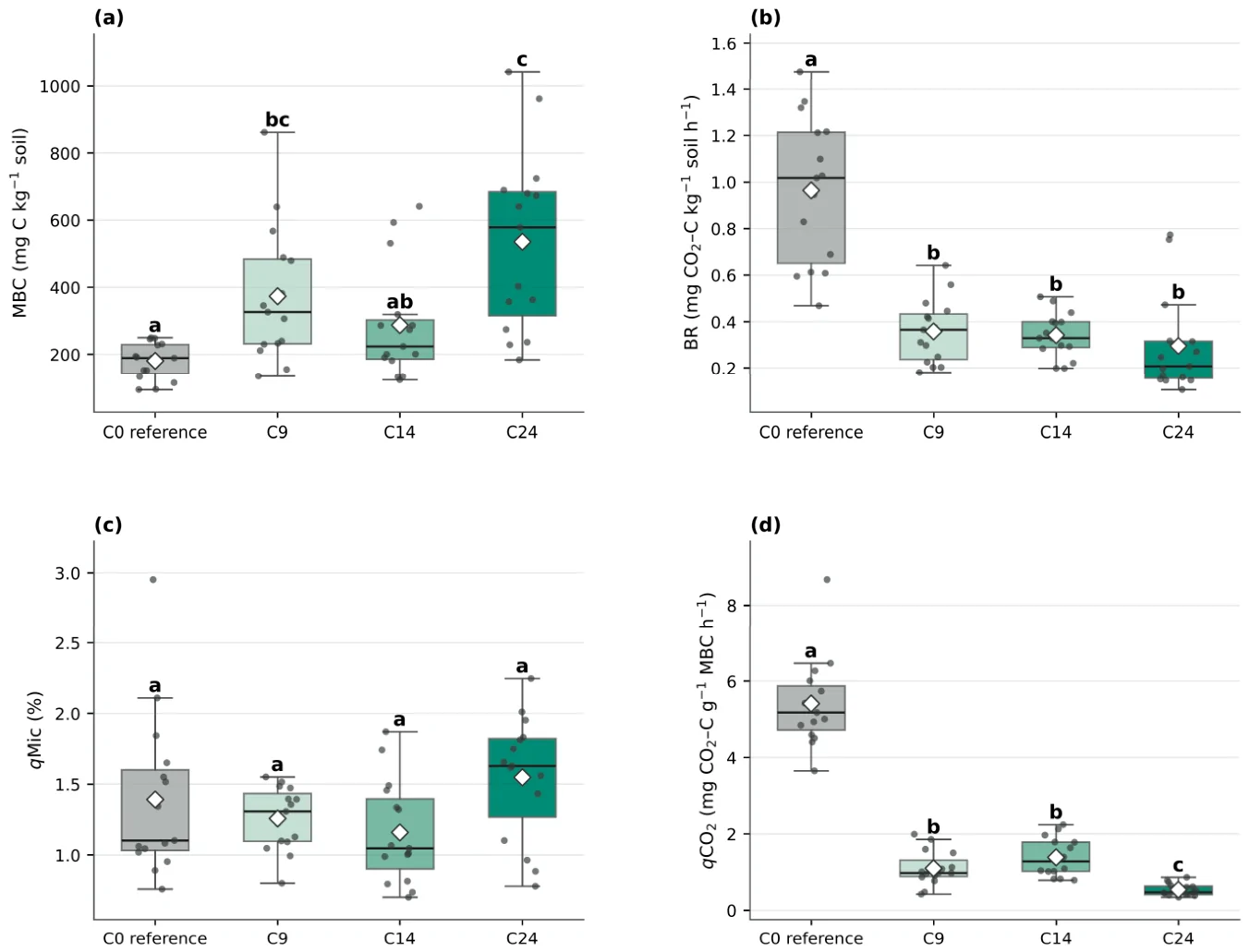
Soil microbial properties across the four chronosequence classes: (**a**) microbial biomass carbon (MBC), (**b**) basal respiration (BR), (**c**) microbial quotient (*q*Mic), and (**d**) metabolic quotient (*q*CO_2_). Boxes show the interquartile range, horizontal lines represent medians, white diamonds indicate means, and jittered points show individual samples (*n* = 15 per class). Whiskers extend to the most extreme observations within 1.5 times the interquartile range. Lowercase letters above the upper whiskers indicate Tukey’s HSD groupings at *p* < 0.05; groups sharing at least one letter are not significantly different. Statistical tests were performed on log_10_-transformed data, although values are displayed on their original scales. All classes have the same letter for *q*Mic because the overall ANOVA was not significant (*p* = 0.0801).

Three of four microbial indicators differed significantly among sites/classes after BH–FDR correction (Table 3; Figure 4). *q*CO_2_ showed the largest effect (ω^2^ = 0.865), followed by BR (ω^2^ = 0.566) and MBC (ω^2^ = 0.337). *q*Mic was non-significant (raw and BH–FDR *p* = 0.0801; ω^2^ = 0.064).

MBC differed among sites/classes (log_10_-scale ANOVA, *p* < 0.001, ω^2^ = 0.337). C9 and C24 each exceeded C0; C14 did not differ from C0 or C9, but C24 exceeded C14 (Tukey *p* = 0.0051). C9 and C24 did not differ significantly (*p* = 0.2005). BR also differed (log_10_-scale ANOVA, *p* < 0.001, ω^2^ = 0.566): C0 exceeded all three afforested sites, which did not differ from one another.

#### Microbial Biomass-Specific Indices

*q*CO_2_ differed strongly among sites/classes (log_10_-scale ANOVA, *p* < 0.001, ω^2^ = 0.865), with C0 highest and C24 lowest; C9 and C14 did not differ. By contrast, *q*Mic did not differ significantly (log_10_-scale ANOVA, raw *p* = BH–FDR *p* = 0.0801, ω^2^ = 0.064), and all sites/classes shared the same compact-letter group.

Overall, the afforested sites were characterized by higher MBC, lower BR, and substantially lower *q*CO_2_ than the degraded reference site, whereas *q*Mic showed comparatively little variation.

### 3.3. Integrated Multivariate Patterns

Using the 11-variable matrix, multivariate composition differed among sites/classes (PERMANOVA Pseudo-F = 16.788, R^2^ = 0.473, *p* = 0.0001; Table 4). PERMDISP was non-significant (F = 1.884, permutation *p* = 0.1454), providing no evidence that the result was driven solely by unequal within-group dispersion. All six pairwise PERMANOVA comparisons remained significant after Holm correction. C0–C24 showed the greatest separation (R^2^ = 0.507, adjusted *p* = 0.0012), whereas C9–C14 showed the smallest (R^2^ = 0.114, adjusted *p* = 0.0116).

**Figure 5:**
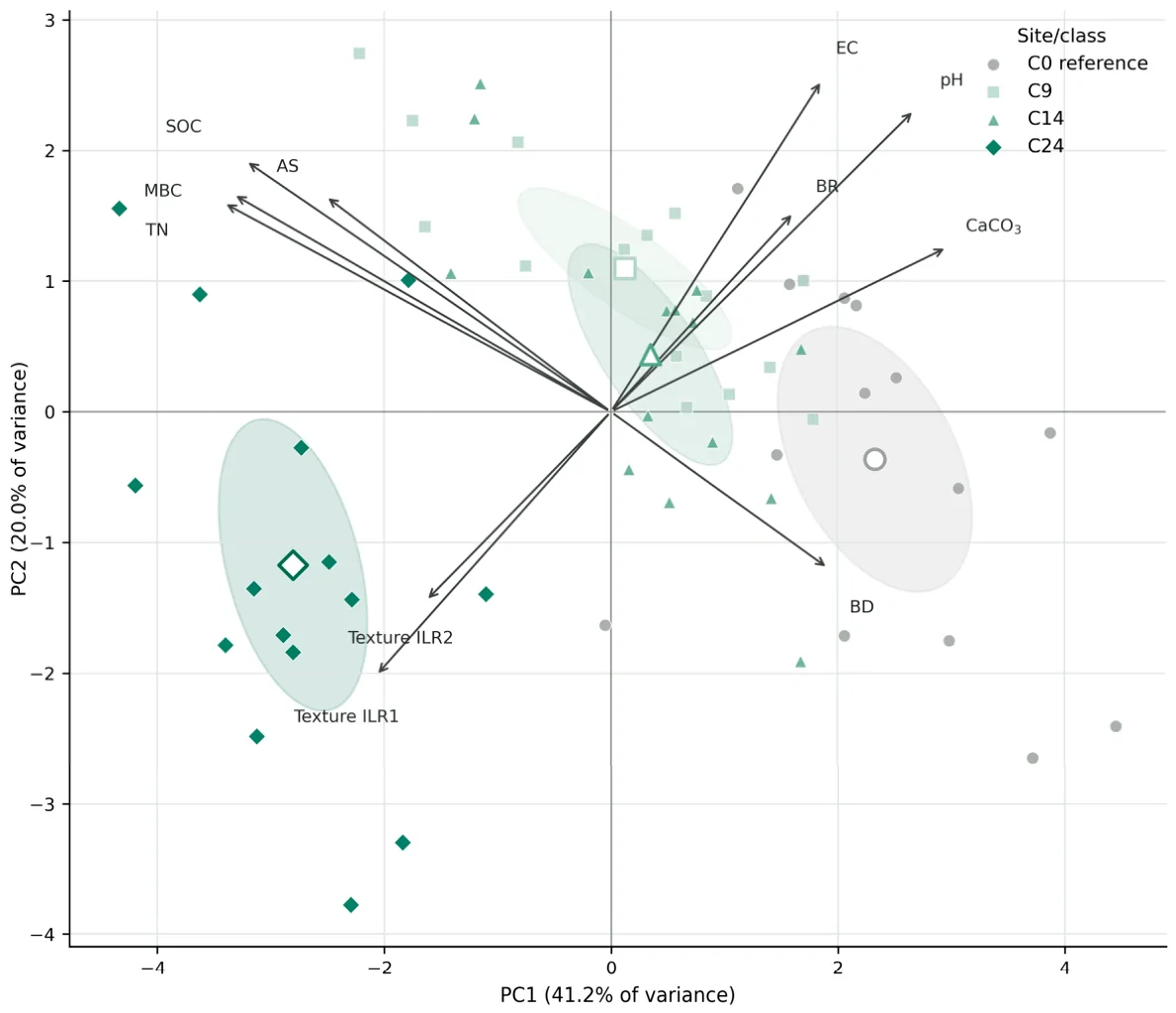
PCA biplot based on the corrected, transformed, and z-standardized 11-variable soil matrix. Points represent individual samples (*n* = 15 per site/class), shaded areas show the 95% group ellipses, and outlined symbols indicate group centroids. Arrows represent variable loadings, with their directions and lengths indicating the direction and strength of association with the principal components. PC1 and PC2 explained 41.2% and 20.0% of the total variance, respectively, accounting for 61.2% cumulatively. Texture ILR1 and ILR2 are the two isometric log-ratio coordinates used to represent the compositional structure of sand, silt, and clay.

PCA summarized the same corrected matrix (Figure 5). PC1 explained 41.2% and PC2 20.0% of variance (61.2% cumulatively). C0 and C24 occupied opposite regions of the ordination, while C9 and C14 were intermediate and partly overlapping. SOC, TN, aggregate stability, and MBC loaded toward C24; pH, CaCO_3_, EC, and BR loaded toward the opposite side. This ordination describes site-associated structure and is not a causal temporal trajectory.

### 3.4. Relationships Among Soil Physicochemical and Microbial Properties

**Figure 6:**
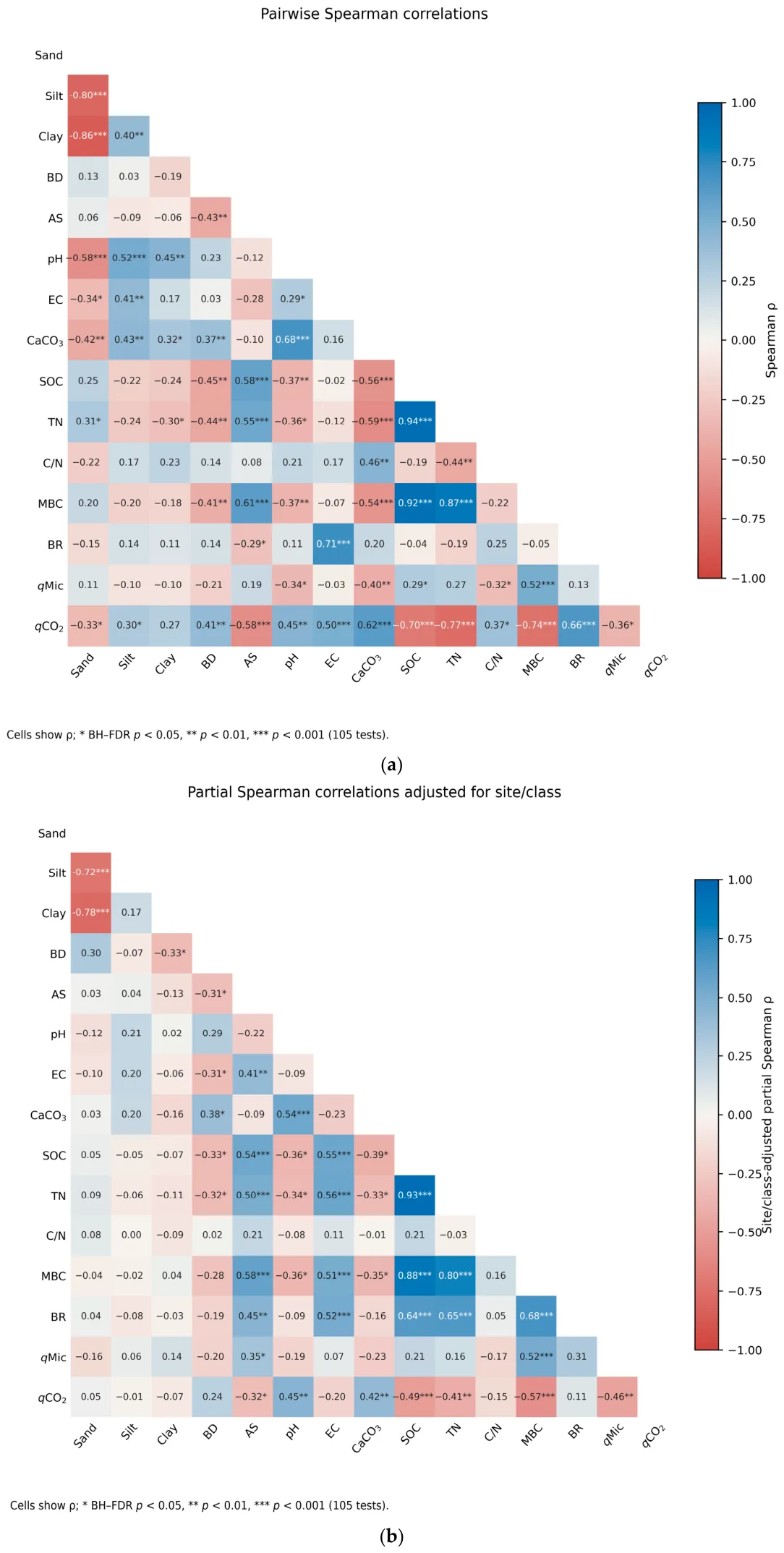
(**a**) Pairwise Spearman correlations among 15 soil variables across all samples (*n* = 60). Cells report Spearman’s correlation coefficient (ρ), with color indicating the direction and strength of each relationship. Asterisks denote significance after Benjamini–Hochberg false-discovery-rate correction across all 105 pairwise tests (* *p*_FDR < 0.05, ** *p*_FDR < 0.01, and *** *p*_FDR < 0.001). Correlations involving sand, silt, and clay should be interpreted descriptively because the three fractions are compositionally constrained to sum to 100%. Relationships involving C/N, *q*Mic, *q*CO_2_, and their component variables should also be interpreted cautiously because mathematical coupling can create or strengthen apparent associations. (**b**) Site/class-adjusted partial Spearman correlations among 15 soil variables (*n* = 60, df = 55). Correlations were calculated from the residuals obtained after regressing the midranks of each variable on indicator covariates for C9, C14, and C24, with C0 serving as the reference class. Cells report the adjusted correlation coefficients, and asterisks indicate significance after Benjamini–Hochberg false-discovery-rate correction across all 105 pairwise tests (* *p*_FDR < 0.05, ** *p*_FDR < 0.01, and *** *p*_FDR < 0.001). Relationships involving C/N, *q*Mic, *q*CO_2_, and their component variables should be interpreted cautiously because of mathematical coupling. Correlations among sand, silt, and clay are also descriptive because these fractions are constrained to sum to 100%.

Pairwise Spearman analysis showed strong positive associations among SOC, TN, and MBC (SOC–TN ρ = 0.936; SOC–MBC ρ = 0.923; TN–MBC ρ = 0.873; BH–FDR *p* < 0.001). pH was positively associated with CaCO_3_ (ρ = 0.680) and negatively associated with SOC (ρ = −0.372), TN (ρ = −0.360), and MBC (ρ = −0.374). Across 105 pairs, 54 remained significant after BH–FDR correction (Figure 6a).

After removing site/class mean differences with three indicator covariates, SOC–TN (partial ρ = 0.931), SOC–MBC (0.881), TN–MBC (0.798), and pH–CaCO_3_ (0.544) remained strong. Thirty-nine of 105 partial correlations remained significant after BH–FDR correction (Figure 6b).

These partial correlations describe residual sample-level associations after adjustment for site/class; they do not isolate a causal stand-age effect. Associations involving C/N, *q*Mic, *q*CO_2_, and their components may be strengthened by mathematical coupling, and relationships among sand, silt, and clay are constrained by their 100% closure.

### 3.5. Stand Age Versus Substrate

**Figure 7:**
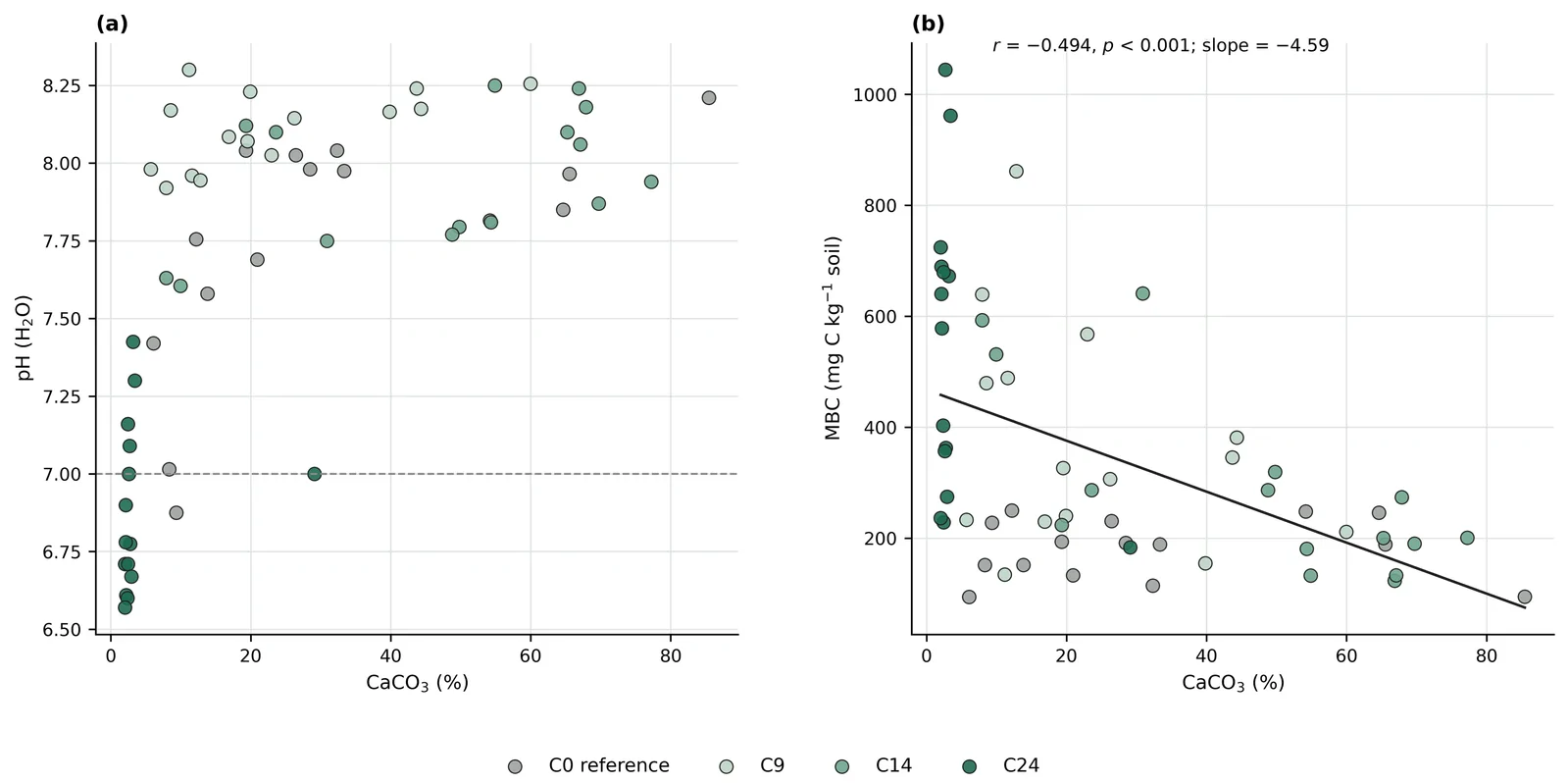
Relationships between calcium carbonate (CaCO_3_) and selected soil properties across the four site/classes: (**a**) pH versus CaCO_3_, with the horizontal dashed line marking neutral pH (pH 7), and (**b**) microbial biomass carbon (MBC) versus CaCO_3_. The solid line in panel (**b**) represents the linear regression fitted to all 60 samples (Pearson r = −0.494, *p* < 0.001). The estimated slope indicates an average decrease of 4.59 mg C kg^−1^ soil in MBC for each one-percentage-point increase in CaCO_3_. This pooled relationship is descriptive and should not be interpreted as evidence of causality.

C24 occupied a distinct carbonate–pH region (Figure 7a), and CaCO_3_ was negatively correlated with MBC across all samples (Pearson r = −0.494, *p* < 0.001; Figure 7b). The marked texture contrasts are more plausibly interpreted as pre-existing site heterogeneity than as change caused by 24 years of plantation development.

Sand, silt, and clay were not entered as separate ANCOVA covariates because their constant-sum constraint makes them linearly dependent. Texture was represented by ilr coordinates in multivariate analysis and examined descriptively in the site/class-adjusted correlation analysis. The exploratory ANCOVA retained measured CaCO_3_ and pH only to test sensitivity to these two observed gradients; it cannot remove unmeasured site differences or separate site identity from plantation age.

**Figure 8:**
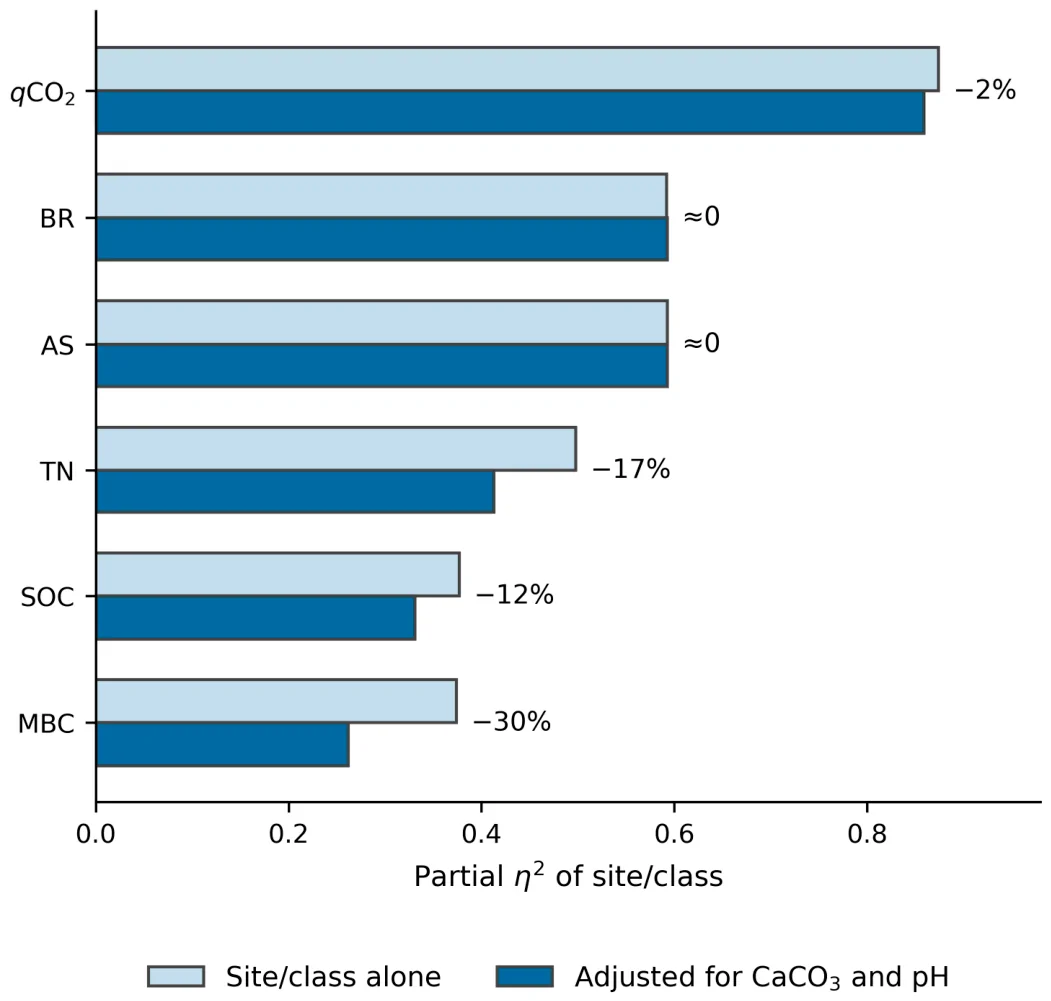
Exploratory comparison of the partial η^2^ associated with site/class before and after adjustment for soil pH and CaCO_3_. Light and dark bars represent unadjusted and covariate-adjusted site/class effect sizes, respectively. Percentage labels indicate the relative change in partial η^2^ after adjustment. The common-slope assumption was violated for MBC, BR, and *q*CO_2_; their results are displayed solely for diagnostic transparency and should not be interpreted inferentially. Covariate adjustment does not separate plantation age from site identity because each nominal age class is represented by a single site. AS, aggregate stability; TN, total nitrogen; SOC, soil organic carbon; MBC, microbial biomass carbon; BR, basal respiration; *q*CO_2_, biomass-specific respiration.

Table 5 and Figure 8 retain the previously reported common-slope ANCOVA outputs solely as transparent exploratory diagnostics. The joint site/class × pH and site/class × CaCO_3_ interaction test rejected homogeneous slopes for log_10_(MBC), log_10_(BR), and log_10_(*q*CO_2_); therefore, their adjusted means and common-slope site/class effects are not used for inference. Homogeneous slopes were not rejected for aggregate stability, log_10_(TN), or log_10_(SOC), but these models still cannot distinguish age from site because each class corresponds to one site.

The six site/class × covariate terms were jointly significant for log_10_(MBC), F(6, 48) = 6.128, *p* < 0.001; log_10_(BR), F(6, 48) = 2.676, *p* = 0.0254; and log_10_(*q*CO_2_), F(6, 48) = 4.840, *p* < 0.001. They were non-significant for aggregate stability (*p* = 0.137), log_10_(TN) (*p* = 0.0844), and log_10_(SOC) (*p* = 0.152). In the modeled raw scale, pH and CaCO_3_ were correlated (Pearson r = 0.603, *p* < 0.001; Spearman ρ = 0.680, *p* < 0.001). Additive-model VIFs were 5.69 for pH and 2.23 for CaCO_3_, and the standardized-design condition number was 4.83. These diagnostics, together with incomplete covariate overlap among sites, preclude interpreting ANCOVA as separation of age from substrate.

### 3.6. Tree Growth

Trees in the 24-year stand averaged 16.94 cm in diameter and 7.00 m in height, roughly double the diameter of the two younger stands (Table 6). Growth was not monotonic with age: the 14-year stand had both the smallest diameters (8.48 cm) and the shortest trees (4.08 m), below those of the younger 9-year stand, consistent with the higher carbonate content and lower organic carbon at that site.

## 4. Discussion

### 4.1. Soil Structural Patterns Across Chronosequence Sites

The clearest physical difference between the afforested sites and the degraded reference site was higher aggregate stability, accompanied by a modest reduction in bulk density. Importantly, aggregate stability remained relatively consistent in sensitivity analyses accounting for CaCO_3_ and pH, indicating that these two edaphic variables alone did not readily explain the observed site differences. In Mediterranean karst landscapes, where shallow soils and limited access to water increase the risk of runoff and erosion, differences in surface-soil structure can have significant ecological consequences. The higher aggregate stability at the afforested sites is consistent with mechanisms commonly associated with forest establishment, including litter inputs, root proliferation, rhizodeposition, fungal hyphae, microbial binding agents, and reduced surface disturbance [42,48]. These processes can promote aggregate formation and persistence, improve infiltration and erosion resistance, and physically protect organic matter [49,50,51,52]. However, these mechanisms were not measured directly and should therefore be regarded as plausible explanations rather than demonstrated causal pathways.

In contrast, particle-size differences primarily reflect inherent site heterogeneity. The oldest plantation contained substantially more sand and less silt and clay than the other sites, a pattern unlikely to result from forest development over the investigated period. Thus, aggregate stability and bulk density represent potentially responsive soil properties, whereas texture primarily characterizes the underlying edaphic template [53].

### 4.2. Carbon and Nitrogen Context for Integrated Soil Functioning

Differences in SOC and TN among the C0, C9, and C24 sites were previously reported as part of a study focused specifically on soil C and N stocks [30]. In the present study, SOC and TN are therefore used primarily as biogeochemical context for evaluating their relationships with soil structural and microbial indicators. The inclusion of the C14 site and the new measurements of aggregate stability, MBC, BR, *q*Mic, and *q*CO_2_ allow the present analysis to address whether carbon and nitrogen status covaries with broader belowground functional properties.

Higher SOC and TN at the afforested sites are consistent with greater organic-matter inputs and retention associated with litter deposition, root turnover, rhizodeposition, reduced disturbance, and improved aggregation [54]. This interpretation agrees with global evidence showing generally positive SOC responses to afforestation, although their magnitude depends strongly on initial soil conditions, stand age, and aridity [55].

However, these differences cannot be interpreted as stand-age effects independently of site conditions. C24 differed substantially in texture, CaCO_3_, and pH, while C14 did not consistently exhibit higher SOC, TN, or MBC than C9. Exploratory adjustment for CaCO_3_ and pH reduced the partial η^2^ associated with site/class for SOC and TN, but these models cannot distinguish plantation age from site identity. For MBC, the common-slope assumption was violated; therefore, the additive ANCOVA results do not support conclusions about the extent to which these covariates explain the observed site differences. Together, these limitations emphasize that SOC concentration alone should not be interpreted as direct evidence of restoration success.

The contrast between C24 and the younger plantation sites should not be interpreted simply as the endpoint of a temporal trajectory. C24 occupied a distinct edaphic setting characterized by sandier soil, lower CaCO_3_, lower pH, and greater geographic separation. Its mean slope was 40%, the same as C9, compared with 30% at C0 and 10% at C14. Its comparatively high SOC, TN, and MBC therefore represent a combined site- and chronosequence-associated pattern. Because plantation age and site identity are confounded, the present design cannot determine whether these differences arose from longer plantation development, more favorable substrate conditions, or their combined influence.

### 4.3. Microbial Biomass and Biomass-Specific Respiration

MBC was higher at the afforested sites and strongly associated with SOC, consistent with greater substrate availability as one possible explanation. However, this relationship does not demonstrate causality. In contrast, the degraded reference exhibited approximately 2.7–3.3 times greater BR despite lower SOC and MBC, indicating that substrate availability alone cannot explain the respiration pattern. Higher MBC under afforestation is nevertheless consistent with previous evidence of increased microbial biomass following reforestation [56,57].

The high BR of the degraded reference remains mechanistically unresolved. Differences in maintenance respiration, substrate accessibility, microbial community composition, or responses to the standardized incubation could contribute to this pattern [18,58]. Importantly, laboratory BR represents potential heterotrophic respiration under imposed conditions and should not be equated with field CO_2_ efflux or decomposition rates. Confirmation would require repeated respiration measurements and complementary microbial and enzymatic analyses.

The *q*CO_2_ pattern requires cautious interpretation because *q*CO_2_ is calculated from BR/MBC and is not an independent response or a direct measure of carbon-use efficiency. Under the standardized incubation, biomass-specific respiration was lower at the afforested sites, but the physiological mechanism cannot be resolved from BR and MBC alone. *q*Mic likewise shares MBC and SOC mathematically and should be interpreted as a relative allocation indicator rather than an independent microbial property. Its omnibus test was non-significant (raw and BH–FDR *p* = 0.0801), indicating that no statistically detectable difference among the four sites was found under the specified analysis. This result does not establish equivalence among sites or the absence of an ecological response. Because *q*Mic expresses the proportion of SOC held in microbial biomass, broadly proportional differences in MBC and SOC can yield smaller differences in their ratio. This provides a possible explanation for the non-significant qMic result despite substantial differences in its component variables, without demonstrating that microbial carbon allocation was unchanged.

### 4.4. Integrated Soil Trajectories Across the Chronosequence

Multivariate analyses revealed coordinated differences among sites, with SOC, TN, aggregate stability, and MBC associated with the afforested portion of the ordination, whereas CaCO_3_, pH, EC, and BR characterized other portions of the multivariate space. However, C9 and C14 partially overlapped and several variables did not vary monotonically with nominal stand age. The ordination therefore represents coordinated site-associated differences superimposed on edaphic heterogeneity rather than a deterministic age-driven trajectory.

The 14-year site is particularly informative in this respect. Although intermediate in nominal age, it did not occupy an intermediate position for several properties: it had the highest mean CaCO_3_ concentration (47.56%) and C/N ratio, its SOC, TN, and MBC did not differ significantly from those of the degraded reference site, and its trees had the lowest mean diameter and height among the three plantations. This departure from a monotonic sequence highlights the importance of considering site heterogeneity when interpreting chronosequence patterns. However, the present design cannot determine the separate contributions of carbonate status and plantation age. Aggregate stability at C14 did not differ significantly from that at C9 or C24. In contrast, qCO_2_ at C14 did not differ significantly from that at C9 but was significantly higher than that at C24 (Table 3). Thus, the observed differences among sites were indicator-specific rather than consistent with a uniform age-related trajectory.

The associations among aggregate stability, SOC, TN, and MBC are consistent with potential interactions among organic-matter inputs, microbial growth, and aggregate formation [59], although these mechanisms were not directly tested. More broadly, the contrasting responses among indicators demonstrate the value of integrating structural, biogeochemical, and microbial measurements rather than relying on SOC alone to characterize belowground soil condition.

Overall, the multivariate response therefore reinforces the value of integrated soil assessment: no single indicator fully captured restoration status, whereas the combined structural, biogeochemical, and microbial dataset clearly distinguished degraded from afforested soils.

### 4.5. Implications for Restoration Monitoring and Management

The study classes represent different sites rather than repeated measurements of the same stand over time, meaning that stand age is completely confounded with site identity. The distinctive particle-size distribution, CaCO_3_ content and pH of C24 demonstrate that complete edaphic equivalence was not achieved. Consequently, the present study cannot attribute all differences between C0, C9, C14 and C24 exclusively to stand age. The covariate analyses therefore should not be regarded as a correction that removes site effects. Their purpose was narrower: to determine whether selected soil responses remained evident after accounting for two measured edaphic gradients, CaCO_3_ and pH. Other site properties—notably slope, effective soil depth, coarse-fragment abundance, and local water availability—may also have influenced the observed differences and remain unresolved sources of confounding.

Several site-associated patterns remained prominent, including higher aggregate stability and lower BR and qCO_2_ at the afforested sites. However, significant site/class-by-covariate interactions precluded interpreting the common-slope ANCOVA results for MBC, BR, and qCO_2_. Moreover, adjustment for measured covariates cannot resolve the confounding between plantation age and site identity. The results therefore support an integrated characterization of the four investigated sites, whereas the generality and direction of temporal changes require replicated chronosequences or longitudinal monitoring.

The findings emphasize that restoration monitoring in Mediterranean karst should not rely solely on tree establishment or SOC. Aggregate stability and microbial measurements can provide complementary information on belowground soil condition [60,61], although *q*CO_2_ should be interpreted specifically as an operational measure of biomass-specific respiration rather than a validated indicator of restoration progress.

The results support the use of *C. libani* afforestation as a restoration tool for degraded, high-elevation karst landscapes. However, they do not suggest that all karst sites will follow the same trajectory. Site quality should therefore remain central to plantation planning. It may be as important to match restoration strategies to local soil depth, carbonate status, moisture availability and parent-material conditions as it is to consider stand age when determining the magnitude of soil carbon recovery [51,62].

Site quality must also be considered when comparing plantations. Differences in carbonate status, texture, soil depth, coarse fragments, slope, and water availability may influence soil properties independently of plantation development. Monitoring programs should therefore combine functional soil indicators with baseline edaphic characterization. Future studies should prioritize replicated chronosequences or permanent plots, seasonal sampling, deeper soil horizons, and measurements of root and litter inputs, microbial communities, and extracellular enzyme activities.

### 4.6. Study Limitations and Future Perspectives

The principal limitation is the lack of independent stand-level replication. Each chronosequence class was represented by a single plantation or reference site; therefore, the 15 sampling locations characterize within-site variability but do not constitute independent replicates of stand age. Consequently, differences among C0, C9, C14, and C24 should be interpreted as site-specific chronosequence-associated patterns rather than unequivocal temporal effects of afforestation. This limitation is particularly relevant for C24, which differed substantially in texture, CaCO_3_, and pH. Although sensitivity analyses evaluated whether selected responses persisted after accounting for CaCO_3_ and pH, they cannot statistically separate stand age from site identity or account for unmeasured factors such as soil depth, coarse fragments, slope-related water redistribution, and microsite moisture availability.

The study was additionally restricted to the upper 0–10 cm mineral soil and a single October sampling campaign. Thus, the results cannot characterize deeper-soil responses or seasonal variability. Microbial measurements were also limited to biomass and respiration-based indices and do not resolve microbial community composition or specific decomposition mechanisms. Replicated spatial and temporal studies incorporating deeper soil sampling and complementary microbial measurements are therefore needed to test the generality and mechanisms of the observed patterns.

## 5. Conclusions

*Cedrus libani* afforestation sites differed substantially from the degraded reference site in the structural, biogeochemical, and microbial properties of surface soils. Afforested soils generally had higher SOC, TN, aggregate stability, and MBC, whereas basal respiration and *q*CO_2_ were lower. These differences were not monotonic with nominal stand age, and variation in SOC, TN, and MBC was partly associated with local edaphic conditions. The coordinated differences in carbon status, soil structure, and microbial properties indicate that integrating multiple soil indicators provides a more comprehensive characterization of belowground conditions than relying on SOC alone. The lower *q*CO_2_ at the afforested sites indicates lower biomass-specific respiration under the standardized incubation conditions, although the physiological mechanism underlying this pattern remains unresolved.

These findings should be interpreted within the constraints of the observational chronosequence design. Each nominal age class was represented by a single site, measurements were restricted to the upper 0–10 cm mineral soil, and sampling was conducted once, in October 2024. Consequently, site identity cannot be separated from plantation age, and the observed differences should be regarded as site-specific chronosequence-associated patterns rather than general causal effects of stand development. Replicated chronosequences and long-term repeated measurements across contrasting karst environments are needed to determine whether these patterns represent general temporal responses to *C. libani* afforestation.

## Figures and Tables

**Table 1:** Location, elevation and climate of the four study sites. Climate values are WorldClim 2.1 (1970–2000) extracted at each plot centroid.

Characteristic	Control (C0)	9-Year Plantation (C9)	14-Year Plantation (C14)	24-Year Plantation (C24)
Field trial area	–	Area 3	Area 2	Area 1
Latitude (N)	36°54′13″	36°54′08″	36°54′28″	36°50′38″
Longitude (E)	34°06′52″	34°06′15″	34°07′08″	34°07′48″
Altitude (m a.s.l.)	1670	1675	1690	1655
Mean slope (%)	30	40	10	40
Planting year	–	2015	2010	2000
Approximate age at October 2024 sampling (yr)	Reference	9	14	24
Mean annual T (°C)	8.7	8.5	8.7	9.0
Annual precipitation (mm)	559	559	556	574
Coldest/warmest month (°C)	−2.4/19.4	−2.6/19.2	−2.4/19.4	−1.6/19.6
De Martonne index	30.0	30.2	29.7	30.2
Köppen class	Dsb	Dsb	Dsb	Dsb
Parent material	Limestone	Limestone	Limestone	Limestone
Soil group	Terra rossa	Terra rossa	Terra rossa	Terra rossa
Texture class	Sandy clay loam	Sandy clay loam	Sandy clay loam	Sandy loam
pH class	Slightly alkaline	Moderately alkaline	Moderately alkaline	Neutral
Stem density (trees ha^−1^)	–	2000	2033	1600
Basal area (m^2^ ha^−1^)	–	15.6	14.6	37.7
Soil samples (*n*)	15	15	15	15

Stand ages represent approximate elapsed age at the October 2024 soil-sampling date based on plantation establishment year. Coordinates recomputed from the UTM zone 36 N field records. The four sites differ by only 0.5 °C in mean annual temperature, 18 mm in annual precipitation, and 35 m in elevation—differences smaller than the climate model’s uncertainty. These small differences suggest that broad-scale climatic variation is unlikely to be the primary source of contrasts among chronosequence classes.

**Table 2:** Soil particle-size distribution, physical and chemical properties (0–10 cm) across the *C. libani* afforestation chronosequence.

Variables	References (C0)	C9	C14	C24	Raw *p*	Effect Size
Soil particle-size distribution						
Sand (%)	69.94 ± 9.73 ^a^	65.96 ± 5.97 ^a^	67.37 ± 7.31 ^a^	82.04 ± 5.75 ^b^	<0.001	ω^2^ = 0.409
Silt (%)	11.26 ± 3.98 ^a^	13.78 ± 5.10 ^a^	11.58 ± 5.64 ^a^	6.11 ± 3.42 ^b^	<0.001	ω^2^ = 0.243
Clay (%)	18.80 ± 6.67 ^a^	20.26 ± 3.77 ^a^	21.06 ± 4.41 ^a^	11.86 ± 5.10 ^b^	<0.001	ε^2^ = 0.307
Soil physical properties						
Bulk density (g cm^−3^)	1.35 ± 0.32 ^a^	1.14 ± 0.22 ^ab^	1.16 ± 0.19 ^ab^	1.09 ± 0.23 ^b^	0.0275	ω^2^ = 0.102
Aggregate stability (%)	61.32 ± 4.68 ^a^	77.77 ± 8.70 ^b^	84.94 ± 8.73 ^b^	81.17 ± 8.05 ^b^	<0.001	ε^2^ = 0.510
Soil chemical properties						
pH (H_2_O)	7.75 ± 0.38 ^a^	8.11 ± 0.12 ^b^	7.95 ± 0.22 ^ab^	6.89 ± 0.27 ^c^	<0.001	ε^2^ = 0.620
Electrical conductivity (dS m^−1^)	0.443 ± 0.141 ^a^	0.311 ± 0.053 ^a^	0.197 ± 0.124 ^b^	0.145 ± 0.119 ^b^	<0.001	ε^2^ = 0.532
Soil organic carbon (%)	1.55 ± 0.75 ^a^	2.85 ± 1.20 ^bc^	2.37 ± 0.83 ^ab^	3.35 ± 1.23 ^c^	<0.001	ε^2^ = 0.313
Total nitrogen (%)	0.045 ± 0.020 ^a^	0.093 ± 0.034 ^bc^	0.065 ± 0.019 ^ab^	0.118 ± 0.049 ^c^	<0.001	ε^2^ = 0.544
C/N ratio	33.94 ± 3.67 ^ab^	30.72 ± 3.58 ^ac^	36.49 ± 3.46 ^b^	28.72 ± 3.58 ^c^	<0.001	ω^2^ = 0.391
Calcium carbonate (CaCO_3_, %)	32.02 ± 24.40 ^ab^	23.40 ± 16.28 ^a^	47.56 ± 23.29 ^b^	4.29 ± 6.88 ^c^	<0.001	ω^2^ = 0.640

Table notes: Values are shown as mean ± standard deviation (*n* = 15 per class). Superscript lowercase letters indicate the results of pairwise comparisons. Groups sharing at least one letter do not differ significantly at *p* < 0.05. Tukey’s HSD was used after one-way ANOVA, whereas Dunn’s test with Holm adjustment was used after the Kruskal–Wallis test. The *p* column presents the raw omnibus *p* values. Effect sizes are expressed as ω^2^ for ANOVA and ε^2^ for Kruskal–Wallis tests. All 11 physicochemical variables remained significant after Benjamini–Hochberg false-discovery-rate correction.

**Table 3:** Soil microbial properties (0–10 cm) across the *C. libani* afforestation chronosequence.

Variables	Control (C0)	C9	C14	C24	Raw *p*	Effect Size
Microbial biomass and activity						
MBC (mg kg^−1^)	180.46 ± 54.74 ^a^	373.45 ± 201.40 ^bc^	287.89 ± 167.73 ^ab^	535.93 ± 268.21 ^c^	<0.001	ω^2^ = 0.337
BR (µg CO_2_–C g^−1^ h^−1^)	0.964 ± 0.318 ^a^	0.358 ± 0.138 ^b^	0.342 ± 0.097 ^b^	0.295 ± 0.212 ^b^	<0.001	ω^2^ = 0.566
Microbial metabolic indices						
*q*Mic (%)	1.391 ± 0.577 ^a^	1.257 ± 0.223 ^a^	1.158 ± 0.362 ^a^	1.548 ± 0.436 ^a^	0.0801 (ns)	ω^2^ = 0.064
*q*CO_2_ (mg CO_2_–C g^−1^ MBC h^−1^)	5.413 ± 1.178 ^a^	1.104 ± 0.454 ^b^	1.391 ± 0.497 ^b^	0.541 ± 0.162 ^c^	<0.001	ω^2^ = 0.865

Table notes: Values are shown as mean ± standard deviation (*n* = 15 per class). Superscript lowercase letters indicate Tukey’s HSD pairwise comparisons based on the log_10_-transformed models. Groups sharing at least one letter do not differ significantly at *p* < 0.05. ns means not significant. The *p* column reports raw omnibus *p* values and effect sizes are expressed as ω^2^. *q*Mic was the only microbial variable that did not differ significantly among classes (raw and BH–FDR-adjusted *p* = 0.0801). The microbial quotient was calculated as *q*Mic = (MBC/SOC) × 100, while the metabolic quotient was calculated as *q*CO_2_ = (BR/MBC) × 1000 and is expressed on an hourly basis.

**Table 4:** Multivariate differences in standardized soil physicochemical and microbial properties among the four *Cedrus libani* chronosequence site/classes based on PERMANOVA.

Analysis	Source/Comparison	df	Pseudo-F/F	R^2^	Raw *p*	Adjusted *p*
PERMANOVA	Site/class	3, 56	16.788	0.473	0.0001	0.0001
PERMDISP	Dispersion among groups	3, 56	1.884		0.1454	0.1454
Pairwise PERMANOVA	C0 vs. C9	1, 28	13.356	0.323	0.0002	0.0012
	C0 vs. C14	1, 28	13.600	0.327	0.0002	0.0012
	C0 vs. C24	1, 28	28.831	0.507	0.0002	0.0012
	C9 vs. C14	1, 28	3.589	0.114	0.0116	0.0116
	C9 vs. C24	1, 28	17.886	0.390	0.0002	0.0012
	C14 vs. C24	1, 28	18.089	0.392	0.0002	0.0012

Table notes: PERMANOVA was based on Euclidean distances calculated from the same corrected, transformed, and z-standardized 11-variable matrix used for PCA. Soil texture was represented by two isometric log-ratio coordinates. EC, SOC, TN, CaCO_3_, MBC, and BR were log_10_-transformed before standardization, whereas pH, aggregate stability, and bulk density were retained on their original scales. C/N, *q*Mic, and *q*CO_2_ were excluded to avoid including variables derived from other components of the matrix. The overall PERMANOVA was based on 9999 permutations, while PERMDISP and pairwise PERMANOVA tests used 4999 permutations. *p* values for the six pairwise comparisons were adjusted using the Holm method.

**Table 5:** Exploratory common-slope ANCOVA results and joint slope-homogeneity tests for models including site/class, pH, and measured CaCO_3_. For MBC, BR, and *q*CO_2_, significant site/class-by-covariate interactions indicated that the common-slope assumption was not met. The corresponding additive-model results are therefore retained only for audit transparency and should not be interpreted inferentially.

Response	η^2^ Site/Class Alone	η^2^ Adjusted	Change	Site/Class *p*	CaCO_3_ *p*	Joint Interaction *p*
*q*CO_2_ (log)	0.874	0.859	−2%	<0.001	0.155	0.000617 (violated)
BR (log)	0.592	0.593	≈0	<0.001	0.064	0.0254 (violated)
AS	0.593	0.593	≈0	<0.001	0.599	0.137
TN (log)	0.498	0.413	−17%	<0.001	0.035	0.0844
SOC (log)	0.377	0.331	−12%	<0.001	0.043	0.152
MBC (log)	0.374	0.262	−30%	<0.001	0.010	0.000080 (violated)

Table notes: Partial η^2^ values describe the site/class effect before and after adjustment for pH and measured CaCO_3_. Percentage change indicates the relative change in partial η^2^ after covariate adjustment. Site/class and CaCO_3_ *p* values were obtained from the additive common-slope models. The joint interaction test evaluates the six site/class-by-covariate terms formed by the three site/class indicators and the two covariates, pH and CaCO_3_. A joint interaction *p* value below 0.05 indicates violation of the common-slope assumption. Variables identified as “log” were analyzed after log_10_ transformation.

**Table 6:** Descriptive summary of diameter at breast height (DBH) and tree height (TH) for the sampled trees.

Parameter	Stand Age	*n*	Min.	Max.	Mean ± SE
DBH (cm)	9 years	60	3.0	21.0	9.25 ± 0.48
	14 years	61	2.0	20.0	8.48 ± 0.57
	24 years	48	6.0	24.0	16.94 ± 0.53
TH (m)	9 years	60	1.0	9.0	5.88 ± 0.24
	14 years	61	0.5	8.0	4.08 ± 0.26
	24 years	48	4.5	9.0	7.00 ± 0.19

Table notes: DBH and TH measurements were obtained from 169 trees nested within nine 10 m × 10 m subplots, with three subplots located in each plantation. Values are presented as mean ± SE and describe the sampled trees only. Because individual trees were nested within subplots and the stand-age classes were not represented by independent plantation-level replicates, the observations should not be interpreted as independent evidence of stand-age effects.

## Data Availability

The raw data and analysis scripts supporting the findings of this study are publicly available in Zenodo at DOI: https://doi.org/10.5281/zenodo.22232927.
